# Application of alkaline phosphatase-to-hemoglobin and lactate dehydrogenase-to-hemoglobin ratios as novel noninvasive indices for predicting severe acute pancreatitis in patients

**DOI:** 10.1371/journal.pone.0312181

**Published:** 2024-11-19

**Authors:** Sha-Yan Chen, Xue-Jing Song, Jiong-Tang Lu, Jian-Xin Liang, Hao Ouyang, Wei-Hua Zheng, Jian-Jun Chen, Zheng-Gang Yin, Hui-Min Li, Yong Zhou

**Affiliations:** 1 Department of Laboratory Science, Binhaiwan Central Hospital of Dongguan, Dongguan, Guangdong, China; 2 Dongguan Key Laboratory of Accurately Etiological Research on the Pathogenesis of Inflammation and Cancer, Dongguan, Guangdong, China; 3 Central Laboratory, Binhaiwan Central Hospital of Dongguan, Dongguan, Guangdong, China; Hokkaido University: Hokkaido Daigaku, JAPAN

## Abstract

Preventing the progression of acute pancreatitis (AP) to severe acute pancreatitis (SAP) is crucial for AP patients. The use of clinical parameters in laboratory facilities for predicting SAP can be rapid, efficient, and cost-effective. This study aimed to investigate the predictive and prognostic value of collected clinical detection parameters, such as serum alkaline phosphatase (ALP) and lactate dehydrogenase (LDH) levels, and their ratios, such as ALP-to-hemoglobin (Hb) and LDH-to-Hb ratios, for the prediction of SAP occurrence, complications, and mortality. In all, 50 healthy controls (CON), 455 patients with mild acute pancreatitis (MAP), 127 patients with moderately severe acute pancreatitis (MSAP), and 93 patients with SAP were included in the study. Thirty clinical parameters were collected, measured, compared, and analyzed. The ratios of ALP/Hb and LDH/Hb were subsequently calculated and evaluated with respect to gender and age and whether they could predict SAP occurrence, complications, and mortality. The results revealed that 15 parameters were significant for the prediction of AP. Furthermore, the following 6 indicators are associated with the occurrence of SAP: ALP, ALP/Hb, LDH, LDH/Hb, CRP, and blood glucose. Among these parameters, the predictive abilities of ALP/Hb and LDH/Hb for SAP were most effective. However, they fail to differentiate between MAP and MSAP or gender in SAP patients. Positive correlations were only observed between the levels of these indicators and age at onset of SAP. In addition, receiver operating characteristic (ROC) curves were generated to predict SAP incidence rates, complications, and mortality. The area under the curve (AUC) values for the ALP/Hb ratio ranged from 0.7097 to 0.7837, whereas those for the LDH/Hb ratio ranged from 0.5043 to 0.7778. These findings suggest that ALP and LDH alone or in combination with Hb possess unique predictive characteristics that may serve as independent factors for predicting SAP occurrence, complications, and mortality; of these, the ALP/Hb ratio is especially predictive of SAP mortality.

## Introduction

Acute pancreatitis (AP) is a frequently encountered acute abdominal condition distinguished by acute pancreatic inflammation due to the activation of pancreatic enzymes; its etiology is categorized as alcoholic, biliary lithiasis, metabolic, or other, and each has diverse prognoses that range from full recovery to death from local or systemic complications and organ dysfunction [1]. In the updated Atlanta classification, AP is stratified into mild (MAP), moderate (MSAP), and severe (SAP) categories [2]. SAP is known for its rapid deterioration and high mortality rates. In recent years, its incidence has become increasingly high because of changes in lifestyle and improvements in living standards in China. However, no specific cure has been established. The best treatment strategy is to prevent AP from deteriorating to SAP, to control AP progression, and to avoid complications and death [3]. Hence, sensitive prediction and vigilant monitoring of SAP progression are imperative.

Numerous scoring systems, including the Ranson, the APACHE II, and the sequential organ failure assessment (SOFA), are available for the evaluation of the severity of AP in terms of the risk of complications and mortality. Nevertheless, the practical application and implementation of these scoring systems are intricate and burdensome [4]. Therefore, the identification of simpler, more sensitive, and accurate laboratory indicators for predicting SAP is clinically important. Numerous biomarkers, including the serum procalcitonin level, the neutrophil/lymphocyte ratio (NLR), the platelet/lymphocyte ratio (PLR), the levels of IL-6, IL-8, IL-12, IL-15, and IL-17, and the red cell distribution width (RDW), are used to assess the severity of AP. Nevertheless, none of these indicators was found to optimally predict disease severity [5].

Several studies have reported that zinc poisoning induces AP [6]. ALP, LDH, and Hb are zinc-containing enzymes related to low-grade inflammation [7, 8]. Therefore, we speculate that these three factors are correlated with AP severity. ALP constitutes a group of enzymes that are primarily located in hepatocytes [9]. ALP, which has important biological functions in the body that sustain essential life processes, is strongly attracted to insulin monomers. ALP can enhance structural and chemical changes in the pancreas and specifically acts as a predictive tool in pancreatic cancer [10]. In addition, after AP inflammation storms, the organ that is first impacted is the liver [11]; thus, the ALP level can be used to monitor AP injury. Moreover, ALP is positively correlated with Hb in thrombi [12]. LDH is an enzyme that plays a role in glycolysis and is expressed mainly in the liver, cardiac muscle, kidney, skeletal muscle, and red blood cells [7]; circulating LDH levels can predict the prognosis of diseases such as acute myocardial infarction [13]. LDH has been included in the Ranson scoring system to assess the severity of AP [14]. Hb, the primary protein component of red blood cells, generally remains constant except in cases of blood loss [15]. The level of Hb in the blood circulatory system is related to gender and age and has a distinct individual trait effect. Multiple studies have shown that various ratios involving Hb can eliminate differences between individuals with diverse diseases; for example, the ferritin-to-hemoglobin ratio has been suggested to be a useful marker for assessing the severity of acute disease [16]. Nevertheless, research on the correlation between AP and serum ALP and LDH-to-Hb ratios is scarce. Therefore, the ratios of ALP and LDH to Hb may be a good way to assess progression to SAP in a more objective and accurate manner. This study aimed to evaluate the predictive and prognostic significance of clinical laboratory test parameters, such as the ALP/Hb and LDH/Hb ratios, in the categorization of AP.

## Materials and methods

### Study design

The current investigation received ethical approval (No. NKYY_YXKT_IRB_2021_155_01) from the Ethical Committee of Tianjin Nankai Hospital, Nankai Clinical College, Tianjin Medical University, in accordance with the Declaration of Helsinki. The Ethical Committee of Nankai Clinical College, Tianjin Medical University, granted a waiver of participant informed consent due to the retrospective design of the study. This study was a retrospective cross-sectional analysis that included a cohort of 50 healthy individuals and 675 patients with AP who were admitted to Tianjin Nankai Hospital from August 1, 2018 to December 31, 2020. Their data were accessed on January 15, 2022 using software belonging to the hospital and laboratory information systems at the hospital used to conduct research. Notably, the controls were relatively healthy outpatients without any digestive diseases or AP and were gender- and age-matched to the AP patients.

**Fig 1:**
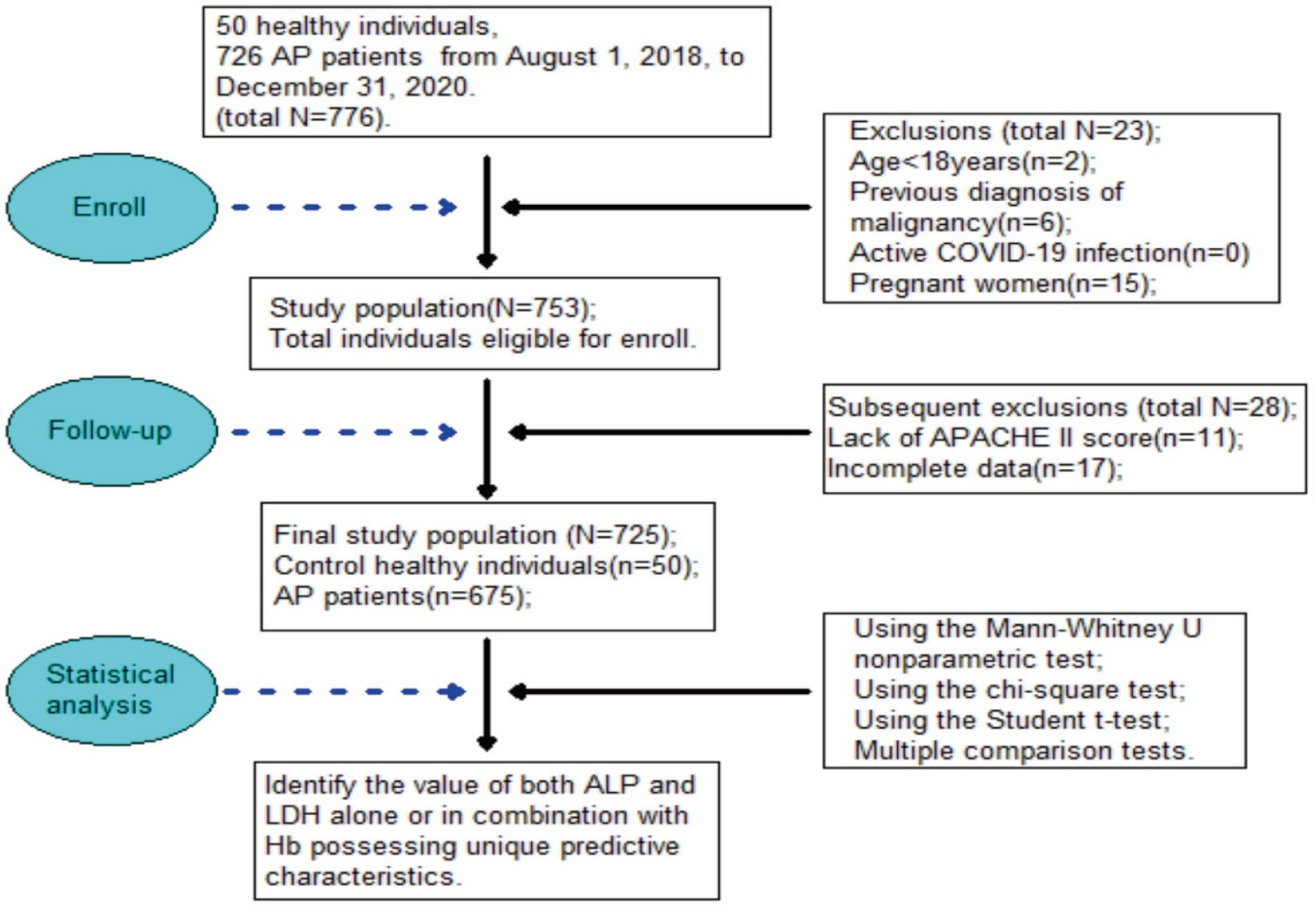
Flow chart of the study. Flow chart of patients screening and inclusion.

In this work, we determined the sample size according to the calculation results of a formula. Healthy controls were selected and matched with AP patients according to gender, age, and the absence of effects of abdominal disease. A flow chart illustrating patient screening and inclusion in this study is shown in Fig 1.

The diagnosis of AP was made when at least 2 of the following 3 criteria were met: characteristic abdominal pain associated with AP, serum amylase and lipase activity levels at least three times higher than the reference limit, and characteristic findings on abdominal computerized tomography scan or transabdominal ultrasound.

The inclusion criteria for this study were as follows: patients were over 18 years of age, had been diagnosed with AP, had available biochemistry and hematology data, and had their serum ALP and LDH levels evaluated in the first blood sample obtained within 24 hours of admission [17].

The exclusion criteria were patients who were younger than 18 years of age, those with a previous diagnosis of malignancy, those with active COVID-19 infection, pregnant women, and patients with missing or inaccessible data.

This study included patients with AP who were categorized according to the International Classification of Diseases, Clinical Modification-10 code K85, as identified by the International Classification of Diseases, Clinical Modification 9th revision code 577.0 21.

The severity of AP was determined to be MAP, MSAP, or SAP via the hospital management information system according to the 2012 Revised Atlanta Classification [1, 11]. In this study, MAP refers to the absence of organ failure and local or systemic complications. Moreover, MSAP is defined as transient organ failure lasting ≤48 hours, with or without local or systemic complications. The mortality rate is less than 8%, and the average hospital stay generally does not exceed two weeks. In cases of SAP, defined as pancreatitis with persistent organ failure (organ failure duration >48 h), with or without local or systemic complications, the mortality rate is significantly higher.

The management of these patients was based on the Revised Atlanta Classification and Management Guidelines put forth by the American College of Gastroenterology Guidelines for AP. Patients exhibiting symptoms of diabetes and hypertension received intravenous insulin infusions, along with treatment including isosorbide dinitrate and urapidil. Plasmapheresis was performed urgently and effectively to treat five patients diagnosed with SAP and hypertriglyceridemia [18].

We analyzed the participants’ demographic characteristics, causes of pancreatitis, any additional medical conditions, and laboratory data of clinical parameters and measurement levels in the first blood sample obtained within 24 hours of admission.

### The collection of clinical data

The hospital information and laboratory information systems at Tianjin Nankai Hospital, Tianjin Medical University, were used to collect and document information related to patient characteristics and laboratory parameters. Documentation included clinical information such as gender, age, etiology of pancreatitis, and comorbidities, as well as laboratory parameters such as fibrinogen (FIB), D-dimer (DD_2_), lymphocytes (lY), neutrophils (N), Hb, hematocrit (HCT), platelets (PLT), alanine aminotransferase (ALT), aspartate aminotransferase (AST), ALP, LDH, total cholesterol (TC), triglycerides (TG), urea nitrogen (Urea), and creatinine (Crea) [19]. The data were derived from measurements made using the first blood sample obtained at initial diagnosis before any medication was administered or treatment was initiated, regardless of the multiple examinations that may have been performed within 24 hours of patient admission. The physiological data (temperature, heart rate, blood pressure, and respiratory rate) were collected at the bedside via time-stamped monitoring records verified by the nurses. We also collected the parameters needed to calculate the following scores: body mass index (BMI), APACHE-II score, BISAP score and SOFA score at symptom onset, which was considered day 0. The fundamental information of the four groups was collected and is presented in Table 1.

**Table 1:** Clinical characteristics of the study participants.

Items	CON	AP	*P* value
Number of participants (number) (male/female)	50(32/18)	675(392/283)	0.576
Age (years)	50.47士21.19	49.12士20.06	0.943
BMI (kg/m^2^)	22.01士2.64	21.90士4.77	0.876
WBC (x10^9^/L)	6.94士3.65	12.10土4.91	0.041
Hypertension history	6(12.00)	5(13.00)	0.565
Diabetes history	4(8.00)	4(10.00)	0.828
Percentage of smokers	5(10.00)	2(10.00)	0.722
FIB (g/L)	3.33(2.00–5.00)	4.21(2.85–8.07)	0.045
DD_2_ (mg/L)	0.30(0–0.80)	0.50(0.32–2.58)	0.190
LY (X10^9^/L)	2.00(1.10–3.20)	2.10(1.23–5.54)	0.854
N (X10^9^/L)	0.30(0.10–0.60)	0.45(0.35–0.78)	0.256
Hb (g/L)	155.00(130.00–175.00)	160.00(133.00–192.00)	0.965
HCT	0.52(0.40–0.80)	0.57(0.47–0.94)	0.832
PLT (X10^9^/L)	146.00(12.005–350.00)	159.00(141.00–528.00)	0.321
ALT (U/L)	28.00(5.00–40.00)	36.00(12.00–187.00)	0.049
AST (U/L)	23.00(10.00–40.00)	27.00(17.00–85.00)	0.059
ALP (U/L)	89.00(40.00–150.00)	126.00(50.00–279.00)	0.038
ALP/Hb	0.60(0.40–0.80)	1.02(0.90–1.42)	0.011
LDH (U/L)	134.00(109.00–245.00)	177.00(125.00–298.00)	0.042
LDH/Hb	1.20(0.90–1.52)	3.12(1.50–5.76)	0.025
TC (mmol/L)	2.59(2.33–5.22)	2.97(2.37–6.58)	0.342
TG (mmol/L)	0.69(0.40–1.70)	1.71(0.50–3.27)	0.167
Urea (mmol/L)	4.89(2.90–8.20)	6.23(3.90–15.72)	0.044
Crea (umol/L)	96.00(46.00–104.00)	201(64.00–354.00)	0.043
CRP (mg/L)	2.47(0–5.00)	12.54(3.00–26.00)	0.034
PCT (ng/mL)	0.02(0–0.05)	2.24(0.02–4.95)	0.031
Blood glucose (mmol/L)	4.80(3.60–6.10)	5.80(4.58–12.39)	0.048
APACHE-II Score	2(0–4)	3(0–4)	0.044
BISAP	0(0–1)	1(0–1)	0.046
SOFA	3(0–4)	5(0–6)	0.039

CON, control; AP, acute pancreatitis; ALP, alkaline phosphatase; ALT, alanine aminotransferase; AST, aspartate aminotransferase; CK, creatine kinase; Crea, creatinine; FIB, fibrinogen; LDH, lactate dehydrogenase; TG, triglycerides; Urea, urea nitrogen; APACHE-Ⅱ, acute physiology and chronic health evaluation Ⅱ; BISAP, bedside index for severity in AP; WBC, white blood cells; SOFA, sequential organ failure assessment; *P* value, CON *vs*. AP.

### BMI assessment

BMI = ㎏/㎡ (㎏: kilograms of weight; ㎡: square meters of height) [17].

### The collection and detection of clinical samples

Blood was collected from control subjects and AP patients who fasted for 8–12 hours within 24 hours of admission. During blood collection, 2 mL of venous blood was obtained from patients lying down. The blood was collected in vacuum tubes containing EDTA-K2 anticoagulant and inert separation glue coagulant, as described in references 13 and 14. The collected samples were combined 8 times each. The tubes containing the inactive adhesive coagulant were subjected to centrifugation at a rate of 3000 revolutions per minute for 10 minutes to avoid hemolysis and hyperlipidemia. After moderate centrifugation, the serum ALT, AST, TC, TG, Urea, Crea, ALP, and LDH levels were analyzed via automated biochemical analysis equipment (Toshiba TBA-2000FR). In addition, the levels of lY, N, HCT, PLT, WBC, and Hb were determined by an automated hematology analyzer (Mindray BC-5390CRP) in blood samples treated with EDTA-K2 anticoagulant. The serum levels of these factors were tested upon admission or within the first 24 hours after admission. The reagents used for these assays were sourced from Mindray and Toshiba. Importantly, our laboratory follows strict internal quality control measures following the International Organization for Standardization (ISO15189) quality management certification for international clinical laboratories. This certification ensures the accuracy and consistency of our laboratory methods in measuring all parameters.

### Statistical analysis

The results are reported as the mean ± standard deviation (SD). For distributions that were not normally distributed, the data are presented as the median (interquartile range) and were compared via the Mann‒Whitney U nonparametric test. Categorical variables are described as counts and percentages and were compared using the chi-square test when appropriate. Two-group comparisons were analyzed using Student’s *t* test, whereas comparisons involving more than three groups were analyzed using one-way analysis of variance with subsequent multiple comparisons tests. All statistical analyses were conducted via the SPSS 16.0 software package (SPSS Inc., Chicago, Illinois, USA) or Prism (GraphPad, La Jolla, California, USA). A *p* value less than 0.05 was considered statistically significant.

## Results

### Clinical characteristics of the study participants

At enrollment, 675 AP patients who met all the inclusion criteria were assessed. To gain a more comprehensive understanding of the characteristics of the study participants in all groups, we analyzed several important clinical parameters. These parameters included BMI (body mass index), WBC count, history of hypertension and diabetes, smoking prevalence, FIB, DD_2_, lY, N, Hb, HCT, PLT, ALT, AST, ALP, LDH, TC, TG, Urea, and Crea. We also considered the APACHE-II, BISAP, and SOFA scores, as mentioned previously. The study participants were categorized into control and AP groups according to their clinical features, as shown in Table 1.

Most of these characteristics did not significantly differ between the two groups; however, significantly lower values were observed for WBC, FIB, ALT, ALP, ALP/Hb, LDH, LDH/Hb, Urea, Crea, CRP, PCT, blood glucose, and APACHE-II, BISAP and SOFA scores in the control group, whereas relatively higher levels were detected in the AP groups, which effectively distinguished the normal population from the AP patients.

### Analysis of significant independent factors between the MAP or MSAP and SAP groups in the cohort

Variables in Table 1 for which *p* < 0.05 were selected and changed to categorical variables. The results indicated that patients’ WBC, FIB, ALT, ALP, ALP/Hb, LDH, LDH/Hb, Urea, Crea, CRP, PCT, blood glucose, and APACHE-II, BISAP and SOFA scores were associated with the differences among the MAP, MSAP and SAP groups (all *p* < 0.05, Table 2).

**Table 2:** Predictors of AP severity in the cohort according to the univariate analysis.

Items	MAP and MSAP	SAP	*P* value
WBC (x10^9^/L)	8.11土3.36	14.25土5.78	0.058
FIB (g/L)	3.22(2.01–7.67)	4.52(2.13–8.45)	0.146
ALT (U/L)	34.00(12.00–185.00)	37.00(10.00–189.00)	0.368
ALP (U/L)	110.00(50.06–252.92)	146.00(63.07–349.62)	0.041
ALP/Hb	0.94(0.76–1.23)	2.35(1.98–2.89)	0.021
LDH (U/L)	176.00(112.00–264.00)	238.00(139.00–327.00)	0.049
LDH/Hb	2.42(1.42–3.73)	4.82(3.83–7.16)	0.035
Urea (mmol/L)	6.37(2.62–17. 32)	9.87(4.95–19.25)	0.149
Crea (umol/L)	178.00(60.32–421.00)	204.00(60.05–583.00)	0.476
CRP (mg/L)	8.83(2.46–25.32)	14.53(6.45–42.54)	0.040
PCT (ng/mL)	1.43(0.01–5.48)	3.26(0.08–7.28)	0.051
Blood glucose (mmol/L)	4.80(3.98–10.01)	7.32(9.58–19.58)	0.048
APACHE-II Score	2(0–4)	3(0–4)	0.073
BISAP	0(0–1)	1(0–1)	0.061
SOFA	8(3–11)	9(3–12)	0.511

MAP, mild acute pancreatitis; MSAP, mild, severe acute pancreatitis; SAP, severe acute pancreatitis; *p value*, MAP and MSAP *vs*. SAP.

As shown in Table 1, the two groups were not significantly different with respect to most of the clinical characteristics. However, patients in the MAP and MSAP groups presented relatively lower ALP, LDH, CRP, and blood glucose levels and lower ALP/Hb and LDH/Hb ratios than patients in the SAP group, whose values were significantly greater. Among these six parameters, the *p* values for ALP/Hb and LDH/Hb were lower, which indicates a stronger ability to differentiate and predict SAP than the other parameters. Importantly, individual differences may be reduced by including the novel Hb ratio in our samples, as Hb values are stable throughout the AP disease process, except for when bleeding occurs. Moreover, Hb levels were evaluated in the first blood sample obtained within 24 hours of admission without the influence of fluid resuscitation.

### Comparison of the ALP/Hb and LDH/Hb ratios among all groups

**Fig 2:**
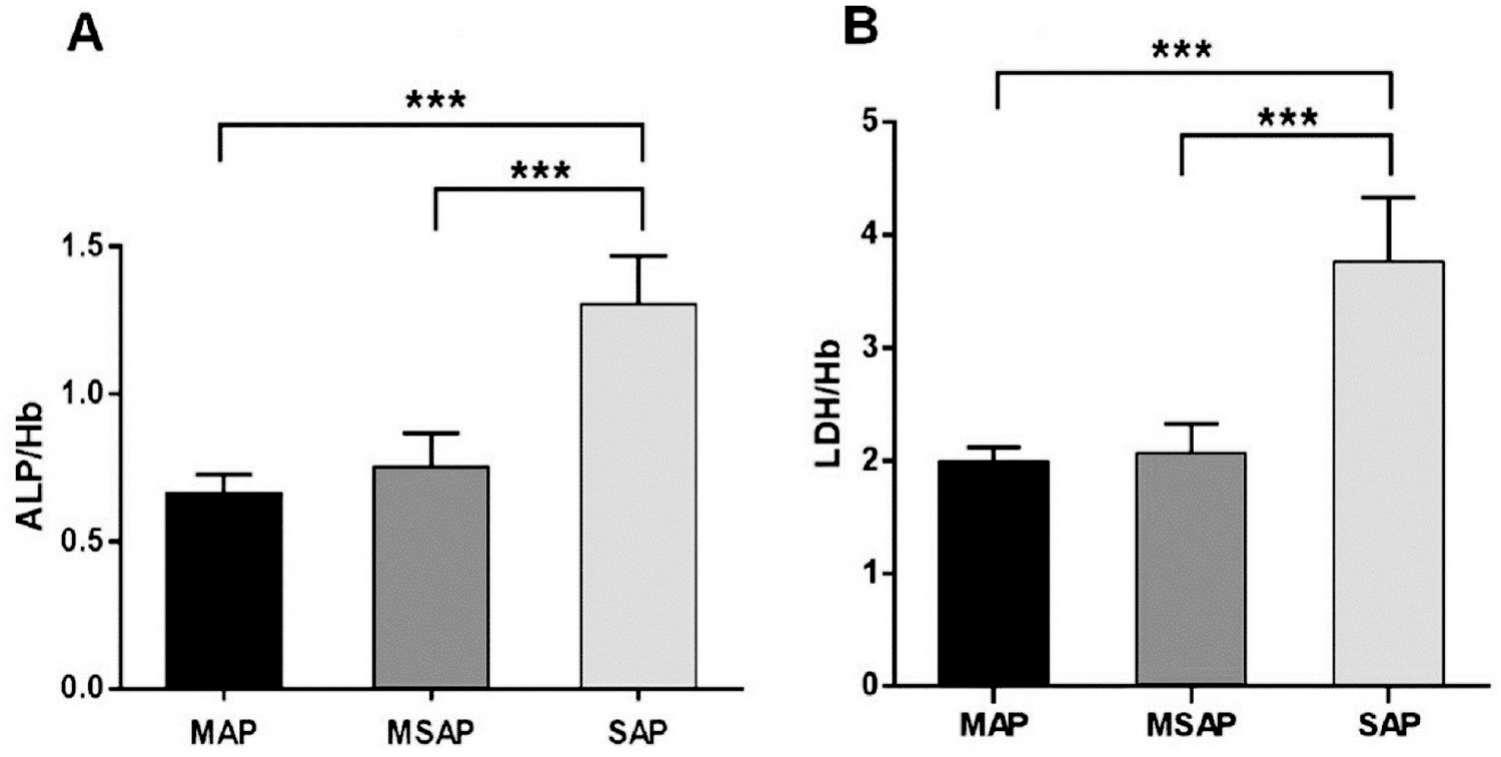
The ratios analysis of ALP/Hb and LDH/Hb in all groups. The ratios of ALP/Hb (A) and LDH/Hb (B) were calculated and had a statistically significant difference among the three groups. All data represented the means ± SD. ***P <0.001*vs* MAP. ***P<0.001 *vs* MSAP.

We then sought to further investigate the characteristics of ALP/Hb and LDH/Hb across all groups. Significant differences were observed across the three groups (*P* < 0.001). Fig 2A shows a substantial increase in the ALP/Hb ratio in the SAP group compared with the MAP and MSAP groups (*P* < 0.001). However, no significant difference was observed between the MAP and MSAP groups. Furthermore, Fig 2B clearly shows that the LDH/Hb ratio in the SAP group was significantly greater than that in both the MAP and MSAP groups (*P* < 0.001). As anticipated, a minimal distinction was observed between the MAP and MSAP groups (*P* > 0.05). Therefore, LDH/Hb exhibited a predictive pattern comparable to that of ALP/Hb in identifying the SAP group rather than the other groups.

### Comparisons of the ALP/Hb and LDH/Hb ratios between female and male SAP patients

**Fig 3:**
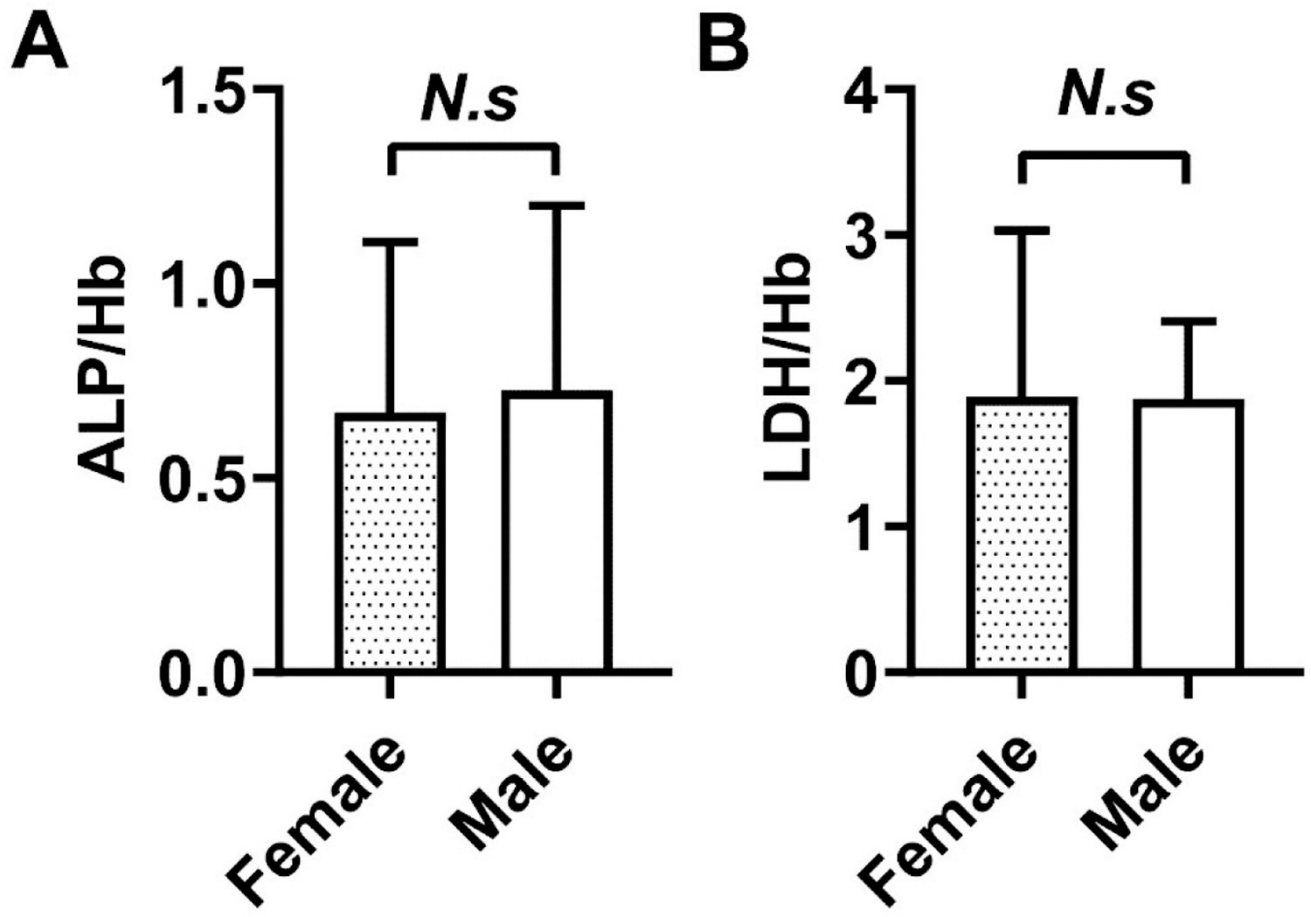
Comparison of serum ALP/Hb and LDH/Hb between female and male patients in the SAP group. Comparison of serum ALP/Hb (A) and LDH/Hb (B) within 24 hrs at their initial visit to the hospital between female and male SAP patients. The serum ALP/Hb and LDH/Hb were detected by an automatic biochemical analysis system and automatic blood cell analyser from three independent experiments. All data were expressed as mean ±SD (*N*.*s*, no significance).

The level of Hb in the blood circulation is related to gender and age, and is generally greater in males than in females. Therefore, we explored the relationship between ratios involving Hb and gender. After the typical ALP/Hb and LDH/Hb ratio patterns were clarified, we further discovered their potential significance in SAP. To address the possible influence of gender, we undertook additional research to examine the unique traits of both females and males. We thoroughly analyzed the ALP/Hb and LDH/Hb ratios, and regrettably, we noticed no statistically significant differences between the two groups (*P*> 0.05; Fig 3).

### Correlations between the ALP/Hb and LDH/Hb ratios and age in SAP patients

**Fig 4:**
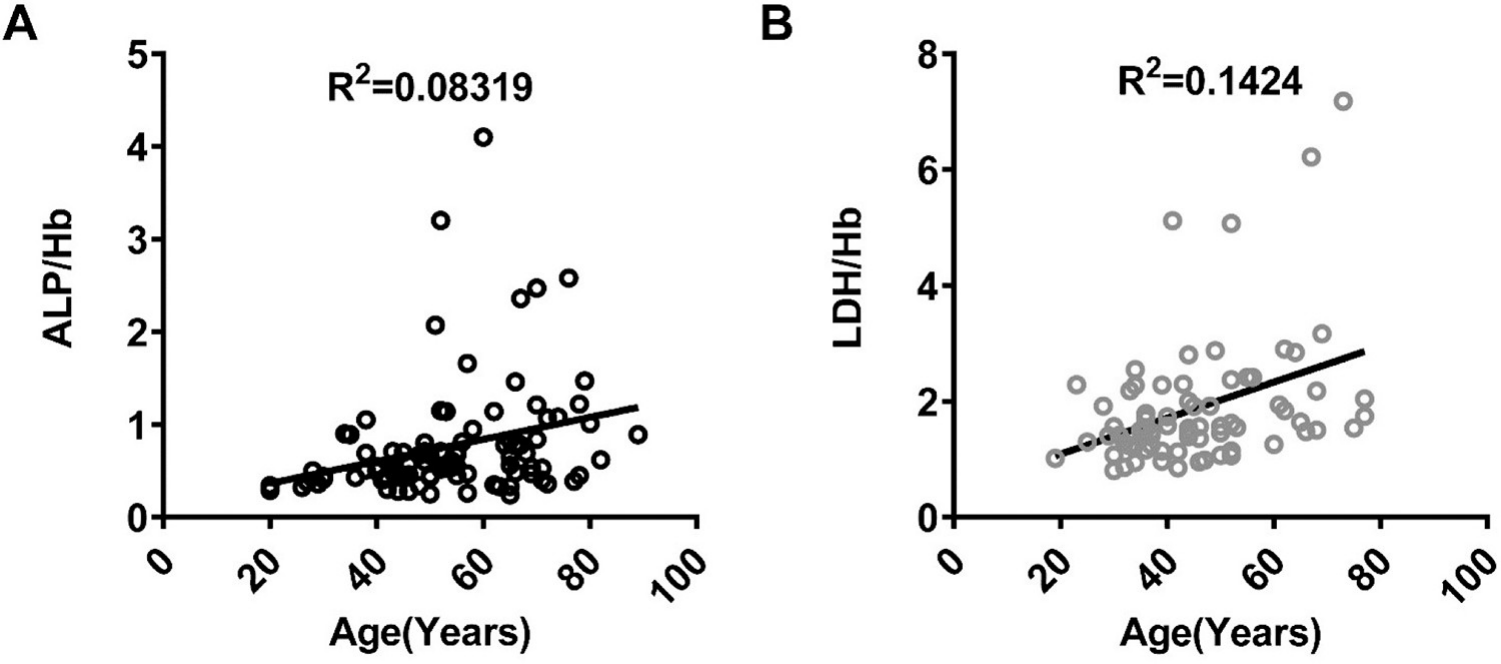
Analysis of serum ALP/Hb and LDH/Hb correlations with age in the SAP group. The analysis of correlations of serum ALP/Hb (A) and LDH/Hb (B) and age at their initial visit to a hospital in the SAP group was shown.

Age is generally understood to be a vital indicator of unfavorable outcomes in various diseases. Physiological metabolism, such as bone growth, pregnancy, growth, maturation and postprandial fat secretion, exerts a considerable influence on the levels of ALP and LDH [12]. Given that age is an indirect measure of physiological metabolism [17], we investigated the associations between the ratios of ALP/Hb and LDH/Hb and age in the SAP group (Fig 4A and 4B). The results revealed certain but not remarkable relationships between these ratios and age in the SAP group. Moreover, the correlation between LDH/Hb and age was more obvious than that between ALP/Hb and age, as shown in Fig 4A and 4B.

### Diagnostic value of the ALP/Hb and LDH/Hb ratios for SAP

**Fig 5:**
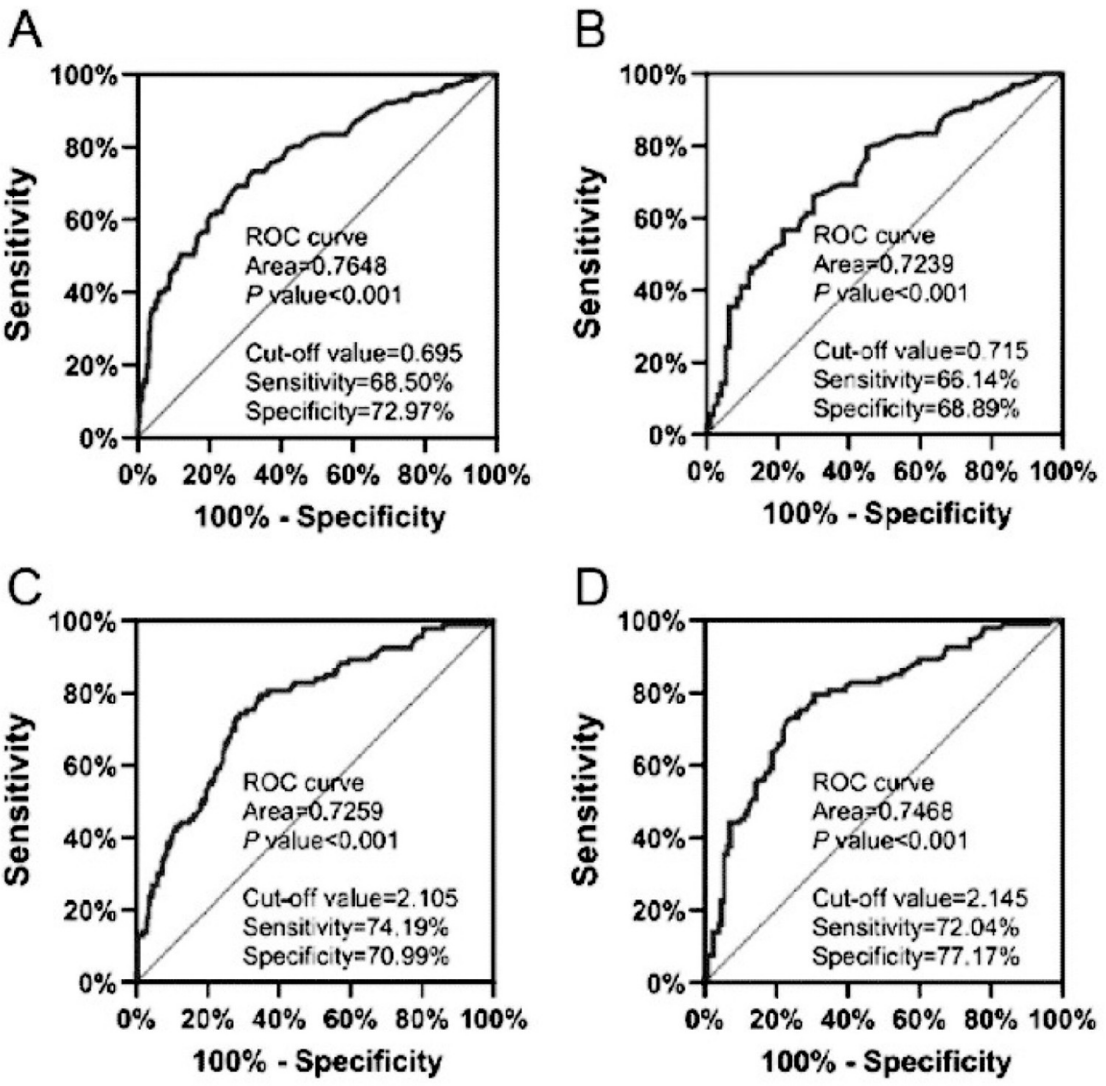
ROC curves were used to evaluate the diagnostic efficacy of ALP/Hb (A-B) and LDH/Hb (C-D) for SAP. The diagnostic efficacy of ALP/Hb (A, B) of SAP patients was evaluated by ROC curves. ROC curves evaluated the diagnostic efficacy of the SAP patients’ LDH/Hb (C, D). AUC, the area under the curve; SAP, severe acute pancreatitis.

The receiver operating characteristic (ROC) curve is usually applied to evaluate predictive performance. AUC > 0.7 was considered useful, whereas an AUC between 0.8 and 0.9 suggested excellent prediction accuracy. The sensitivity and specificity were calculated to compare the prediction ability of the models. To demonstrate the diagnostic efficiency of the ALP/Hb and LDH/Hb ratios in the SAP group, we utilized ROC curves for diagnostic analysis. We compared the ALP/Hb and LDH/Hb ratios between the SAP and MAP groups or the SAP and MSAP groups. The critical value, optimal sensitivity, specificity, and AUC of the ALP/Hb ratio in the SAP group compared with the MAP group were 0.695, 68.50%, 72.97%, and 0.7684, respectively (Fig 5A). However, these values were lower at 0.715, 66.14%, 68.89%, and 0.7239, respectively, when compared with the MSAP group (Fig 5B). Similarly, for the LDH/Hb ratio in the SAP group compared with the MAP group, we obtained a critical value of 2.105, along with an optimal sensitivity of 74.19%, a specificity of 70.99%, and an AUC of 0.7259 (Fig 5C). Those values in the SAP group when compared with the MSAP group were 2.145, 72.04%, 77.17%, and 0.7468, for the critical value, sensitivity, specificity and the AUC, respectively, which are greater than those of the SAP group compared with the MAP group (Fig 5D).

### The ALP/Hb and LDH/Hb ratios predict complications and mortality in SAP patients

**Fig 6:**
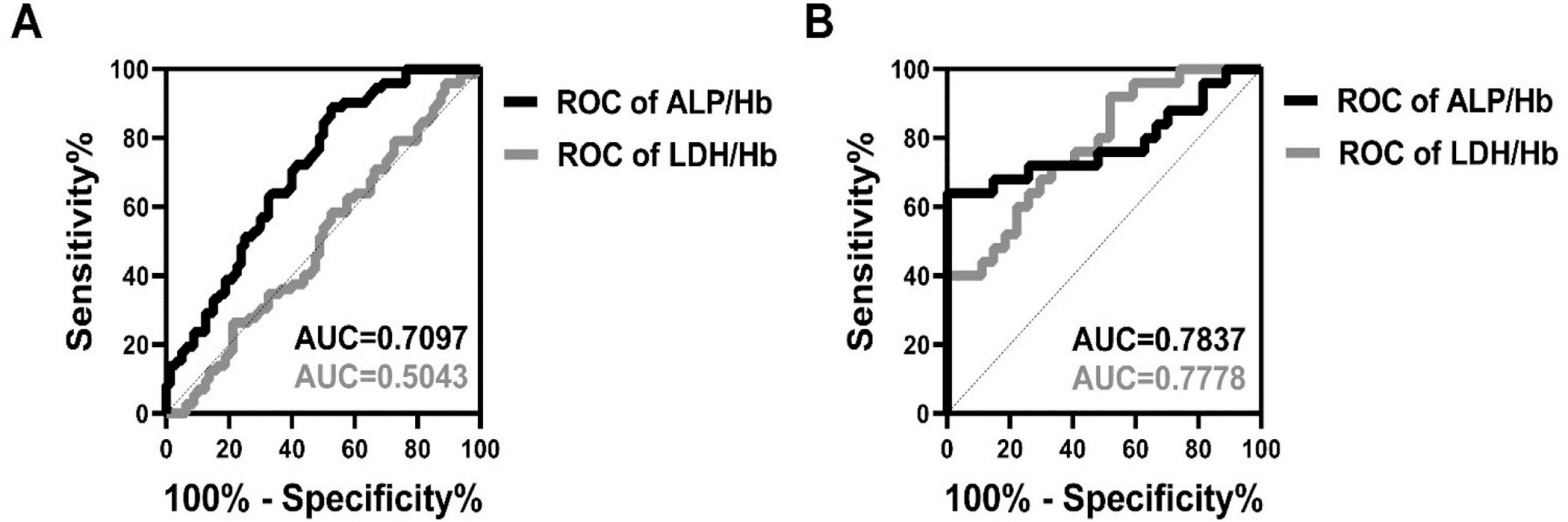
Analysis of prediction of complications (A) and mortality (B) by ALP/Hb and LDH/Hb in the SAP group. The analysis of prediction complications (A) and mortality (B) in SAP by serum ALP/Hb and LDH/Hb was presented. AUC, the area under the curve; SAP, severe acute pancreatitis.

The definition of SAP is commonly recognized to include temporary organ failure, regardless of whether the problems are present only in one affected organ or across the body. The mortality rate is high and often ranges from 20% to 40% [18], especially in SAP patients with infections of the pancreas, in whom the mortality rate increases by 18% [11]. Hence, it is crucial to anticipate the onset of complications and death in SAP patients. We utilized ROC curves to assess the predictive efficacy of the ALP/Hb and LDH/Hb ratios. The findings revealed that AUC for ALP/Hb was 0.7097 for complications and 0.7837 for mortality in SAP patients (as shown with black curves in Fig 6A and 6B). In contrast, LDH/Hb had an AUC of 0.5043 for complications and an AUC of 0.7778 for death (as represented with grey curves in Fig 6A and 6B). These data suggest that the ALP/Hb ratio is a more reliable predictor than the LDH/Hb ratio for assessing prognosis, especially the mortality, rather than comorbidities, of SAP patients.

## Discussion

AP, also known as acute abdomen in clinical terms, is a frequently encountered disease with diverse etiologies that results from the activation of pancreatic enzymes and partly results in systemic inflammatory response syndrome (SIRS) and necrosis of pericardial tissue [1]. Approximately 20–40% of AP cases progress to SAP, which is associated with multiple organ dysfunction syndrome (MODS), sepsis, and high mortality [3, 4]. Currently, the incidence of AP in China is increasing due to improvements in living standards and lifestyle changes. Moreover, completely curative treatments do not yet exist. As a result, the early, rapid, and effective prediction of transformation in AP and SAP patients is clinically challenging [20]. Therefore, it is crucial to explore new markers that can accurately predict the occurrence and development of SAP.

Compared with imaging examinations [2, 5], some clinical laboratory parameters, including coagulant system parameters such as FIB, DD_2_, Hb, HCT, and PLT and biochemical parameters such as ALT, ALP, LDH, TG, and Crea, can be detected more rapidly and more efficiently from blood samples by a laboratory facility at low cost and are the routine test parameters in clinical practice [9, 21]. Nonetheless, the associations between these markers and disease severity remain inconsistent or inconclusive [22, 23]. This research was similar in using parameters such as WBC, FIB, ALT, Urea, Crea, CRP, PCT and blood glucose to classify AP [20]. Others have reported that even if serum amylase and lipase serve as crucial diagnostic AP markers, they still lack consistency in classifying AP [24], which is comparable to our findings. We conclude that clinical laboratory parameters have advantages and limitations, and further research is needed.

Zinc is closely related to AP [6], and ALP, LDH, and Hb are zinc-containing enzymes [25, 26]. Additionally, Hb, like RAW, is a physical parameter of red blood cells [27], is relatively stable except during active bleeding, and can be utilized as a means to reconcile interindividual discrepancies among different patients in the early stage of disease without blood or fluid infusion, as described in previous studies [15]. Many studies have mined the predictive significance of ratios containing RAW [28, 29]. Therefore, we speculate that the abovementioned parameters combined as a new ratio may have the potential to predict AP.

It is widely acknowledged that abnormal ALP levels in the blood can reflect tissue damage or disruption of normal processes in the body and are associated not only with liver function [30], renal function [22], skeletal muscle composition [9], and even inflammatory states but also with bile duct obstruction and zinc levels [12, 17]. Previous studies have demonstrated a positive correlation between elevated levels of ALP and prolonged hospital stays in patients with certain diseases with low levels of inflammation rather than those with diseases with high levels of inflammation [18]; it seems that ALP may be more related to AP than SAP, which is similar to the results of this study. One etiology of AP is pancreatic enzyme activation due to bile duct obstruction [1], and thus a mechanistic basis has been established for elevated ALP. In China, gallstones are universally regarded as the most common etiology of AP [31]. High ALP levels are an important indicator of biliary pancreatitis and can also increase obstructive jaundice. Moreover, some studies report that zinc phosphide ingestion results in AP [8]. Therefore, the high levels of the zinc-containing enzymes ALP and LDH are likely closely related to AP. In addition, continuous monitoring methods can be used to detect ALP, and many factors, such as vigorous exercise before detection and rapid growth in children and pregnant women, may temporarily affect ALP levels [12].

LDH is known to be associated with pancreatitis [32], as the disease mechanism involves LDH, which is an important enzyme of anaerobic glycolysis and gluconeogenesis that is thought to be related to pancreatic cell damage [7]. It has also been reported that pernicious anemia can cause high lactate dehydrogenase levels [33]. Therefore, we can infer that the ratio of LDH to Hb further increases the sensitivity, which is conducive to predicting SAP from the point of view of LDH detection. The principle of LDH detection is that LDH can dehydrogenate lithium lactate, which can be analyzed via colorimetric methods. Hence, pancreatic cell injury, tissue hemorrhage, and necrosis cause intracellular LDH to spill into peripheral blood serum, further increasing its detection level, and thus its *P* value is much smaller than that of other parameters.

Most previous studies on ALP and LDH have focused on the application and development of biomarkers for cancer diagnosis since LDH is associated with the activation of oncogenes such as HIF-α and Myc, as well as with tumor proliferation and metastasis [9, 10, 12, 13]. In patients with head and neck squamous cell carcinoma and in those with prostate cancer, studies have revealed a correlation between these biomarkers and surgical prognosis [34]. In nontumor disease models, clinical studies have shown that ALP and LDH are associated with systemic mastocytosis and have important prognostic significance. An association has also been reported between increased LDH levels and mortality in COVID-19 patients receiving mechanical ventilation, which indicates the prognostic value of LDH in patients with severe infections. However, no studies have explored the ALP-to-Hb ratio or the LDH-to-Hb ratio. Based on the above studies, the ratios of ALP-to-Hb and LDH-to-Hb may have important application value in the diagnosis of various diseases, surgical prognosis assessment and prognostic monitoring, but the specific effects and mechanisms need to be verified and optimized through more clinical studies.

In this study, our examination of 30 clinical parameters revealed no statistically significant differences among the 15 clinical parameters in Table 1. These findings indicate that the characteristics of these clinical parameters support the validity of our study. However, 15 indicators (WBC, FIB, ALT, ALP, ALP/Hb, LDH, LDH/Hb, Urea, Crea, CRP, PCT, blood glucose, and APACHE-II, BISAP, and SOFA scores) were significantly associated with distinguishing the normal population from AP patients (Table 1). Interestingly, the *P* values of ALP, ALP/Hb, LDH, and LDH/Hb were lower than those of the other variables. Therefore, we focused our study on their potential as predictors of AP.

One parameter is AST, an enzyme found in various tissues including in the mitochondria of human cardiomyocytes and hepatocytes [34]. Yang J. *et al*. demonstrated that liver injury occurs in some AP patients and that its intensity is positively associated with the severity of pancreatitis and with a longer disease duration [23]. While inconsistent in this study, the serum AST level in the AP group was not obviously greater than that in the CON group. Moreover, similar to Urea, higher serum levels of Crea are observed in AP patients compared with CON, but rarely in MAP and MSAP patients when compared with SAP patients, which possibly because of the systemic inflammatory response and high catabolic rate, which leads to a considerable increase in creatinine. Simultaneously, activating the sympathetic nervous system causes constriction of the renal arterioles and ultimately leads to a decline in renal function [22]. Chen Y. *et al*. reported that Crea is a reliable indicator of long-term organ failure [35]. Wang J. *et al*. reported that the Crea level is a more appropriate predictor of SAP [36]. Nevertheless, our investigation also confirmed that elevated levels of Crea are associated with AP but not with SAP. TG and TC levels are positively associated with organ failure, persistent renal failure, persistent shock, necrosis, intensive care unit (ICU) admission, and death [9]. Nevertheless, our results were inconsistent with these findings, as were our results regarding FIB, DD_2_, PLT, LY, N, Hb and HCT. Thus, our study aimed to assess the prediction capacity of these factors in SAP patients with high inflammation, which may yield valuable insights and corroborate findings from prior research.

Several scoring systems, such as the Ranson, APACHE II, BISAP, SOFA, and MCTSI, are commonly utilized in clinical practice to distinguish AP patients from healthy individuals [2]. These findings are consistent with the results shown in Table 1 of our paper. However, these systems still cannot predict AP subtypes, especially early predictions of SAP. In this study, the sensitivity of these indicators may have decreased because we used measurements from the blood obtained just after admission, and some patients may have been in the initial disease state and had not progressed or worsened. In addition, these scoring systems pose challenges due to their labor-intensive nature, time-consuming process, and inconvenience for clinical application [5].

To further investigate the clinical significance of the 15 parameters in classifying AP, especially SAP, we compared the levels of these parameters between MAP and SAP and between MSAP and SAP. We found that 6 indicators, ALP, ALP/Hb, LDH, LDH/Hb, CRP, and blood glucose, were associated with the occurrence of SAP (Table 2). Among these 6 parameters, the predictive abilities of ALP/Hb and LDH/Hb were better for SAP. We speculate that this may be because the ratio increases the sensitivity of the prediction.

The clinical predictive value of the ALP/Hb ratio and the LDH/Hb ratio in differentiating AP subtypes is shown by a comparison of the three groups, as seen in Fig 2A and 2B, which revealed that the ALP/Hb and LDH/Hb ratios were considerably greater in the SAP group than in the MAP and MSAP groups (*P* < 0.001). However, no significant difference was observed between the MAP and MSAP groups. These findings suggest that employing ALP/Hb and LDH/Hb ratios in this creative approach holds considerable promise, specifically in the SAP group.

Previous research has indicated potential associations between Hb and gender and between ALP and pregnancy [34], and thus we evaluated the correlations between the ALP/Hb and LDH/Hb ratios and gender in individuals diagnosed with SAP. However, no significant differences were observed in the ALP/Hb or LDH/Hb values between male and female patients (Fig 3). This discovery aligns with prior research on the level of irisin in the blood of healthy individuals, adults with prediabetes, and young individuals with no health issues [17]. Therefore, factors other than muscle content, particularly in males, might have a minimal influence on the regulation of ALP/Hb and LDH/Hb levels in SAP patients. Nevertheless, whether gender has a causal or consequential effect on SAP remains difficult to ascertain.

Furthermore, significant positive associations were reported between ALP and Hb levels and older individuals and children [37]. Age is widely recognized as a crucial and valuable indicator of negative outcomes in various diseases. Numerous widely accepted predictive models in clinical medicine have included age for predicting severity or mortality. As shown in Fig 4, our findings indicated that age was a significant and autonomous factor in SAP patients. However, correlation analyses revealed no statistically significant relationship between ALP or LDH levels and age in obese diabetic patients or in individuals who exercise [24]. Although the correlations of ALP/Hb and LDH/Hb with age in the SAP group were inconspicuous, they indicated favorable linear connections, particularly the substantially greater LDH/Hb correlation in the SAP group. These findings present partial evidence that supports that ALP/Hb and LDH/Hb are independent factors. However, our current understanding of the molecular mechanisms underlying their involvement in SAP is still limited.

To explore the clinical diagnostic value of ALP/Hb and LDH/Hb in SAP, we utilized ROC curves (Fig 5) with AUCs >0.7. According to “Clinical Application Evaluation of Clinical Biochemical Test Items” in the field of laboratory medicine [17], the AUC values of the clinical test parameters are as follows: 0.7< = AUC<0.9 indicates high predictive diagnostic value, which is more common; AUC> = 0.9 indicates high predictive diagnostic value, which is better; AUC = 1: indicates perfect prediction without flaws, and in the vast majority of cases, no ideal prediction of diagnosis exists; 0.5< AUC <0.7: indicates low predictive diagnostic value, which is relatively common. Therefore, our data may be acceptable and with high diagnostic capabilities when compared the ALP/Hb and LDH/Hb ratios between the SAP and MAP groups or the SAP and MSAP groups.

In addition, we also utilized ROC curves to predict the mortality rate and complications associated with SAP, as shown in Fig 6. The results of this study showed that the ALP/Hb ratio, rather than the LDH/Hb ratio, was highly predictive of the mortality rate of SAP patients. Moreover, when predicting the complications of SAP, these two ratios had AUCs >0.7 and were thus highly effective.

Briefly, our findings contained two major clinical highlights. First, this ALP/Hb and LDH/Hb diagnostic SAP model could help simplify clinical management by reducing constant monitoring of patients, which increases their financial burden. Furthermore, we successfully determined the distinctive ALP/Hb and LDH/Hb features in predicting SAP. This allowed us to comprehend the clinical specialization of SAP using an innovative approach, which could assist clinicians in making predictions based on readily available clinical data.

However, this study had several shortcomings. First, we could not initially identify a definitive cause‒and‒effect relationship between the ALP/Hb and LDH/Hb ratios and the effectiveness of clinical treatment. Second, it is crucial to acknowledge that the actual levels of clinical parameters in the circulation can differ according to the specific assay used for assessment in the laboratory science department of the hospital. However, our findings were similar to those reported in numerous earlier investigations. Third, the number of AP patients in our study was insufficient. Although our study had a small sample size, our findings offer useful insights to guide future studies with a larger group of participants to better understand the importance of the ALP/Hb and LDH/Hb ratios in SAP patients. Finally, the data collection was too dependent on the detection methods of the above parameters, and therefore, the statistics may be biased.

## Conclusion

The current investigation revealed the predictive ability of both ALP and LDH levels alone or in combination with Hb, demonstrating their potential as novel biomarkers linked with disease progression and mortality in SAP patients. Thus, the ALP/Hb and LDH/Hb ratios could be useful indicators for predicting the clinical progression and prognosis of SAP patients, and specifically, the ALP/Hb ratio can also predict mortality in SAP patients.

## Supporting information

S1 Dataset (XLSX)

## References

[1] Portelli M, Jones CD. Severe acute pancreatitis: pathogenesis, diagnosis and surgical management. Hepatobiliary Pancreat Dis Int. 2017;16(2):155–9. Epub 2017/04/07. doi: 10.1016/s1499-3872(16)60163-7 . 28381378

[2] Harshit Kumar A, Singh Griwan M. A comparison of APACHE II, BISAP, Ranson’s score and modified CTSI in predicting the severity of acute pancreatitis based on the 2012 revised Atlanta Classification. Gastroenterol Rep (Oxf). 2018;6(2):127–31. Epub 2018/05/22. doi: 10.1093/gastro/gox029 ; PubMed Central PMCID: PMC5952961. 29780601 PMC5952961

[3] McIver W. Aggressive or Moderate Fluids in Acute Pancreatitis. N Engl J Med. 2022;387(23):2199. Epub 2022/12/09. doi: 10.1056/NEJMc2213440 . 36477045

[4] Tenner S, Vege SS, Sheth SG, Sauer B, Yang A, Conwell DL, et al. American College of Gastroenterology Guidelines: Management of Acute Pancreatitis. Am J Gastroenterol. 2024; 119(3):419–437. Epub 2023/11/07. doi: 10.14309/ajg.0000000000002645 . 38857482 PMC13221274

[5] Kothari S, Kalinowski M, Kobeszko M, Almouradi T. Computed tomography scan imaging in diagnosing acute uncomplicated pancreatitis: Usefulness vs cost. World J Gastroenterol. 2019;25(9):1080–7. Epub 2019/03/14. doi: 10.3748/wjg.v25.i9.1080 ; PubMed Central PMCID: PMC6406186. 30862996 PMC6406186

[6] Wang M, Phadke M, Packard D, Yadav D, Gorelick F. Zinc: Roles in pancreatic physiology and disease. Pancreatology. 2020;20(7):1413–20. Epub 2020/09/13. doi: 10.1016/j.pan.2020.08.016 ; PubMed Central PMCID: PMC7572834. 32917512 PMC7572834

[7] Desai AD, Chinta S, Yeh C, Shah VP, Shah R, Paskhover B, et al. An analysis of lactate dehydrogenase (LDH) levels in advanced stage IV melanoma of the skin: prognostic capabilities and demographic variability. Arch Dermatol Res. 2023;315(4):799–806. Epub 2022/11/02. doi: 10.1007/s00403-022-02425-0 . 36318305

[8] Bhandari R, Basnet K. A case report on zinc phosphide ingestion resulting to acute pancreatitis. Ann Med Surg (Lond). 2022;78:103859. Epub 2022/06/24. doi: 10.1016/j.amsu.2022.103859 ; PubMed Central PMCID: PMC9207063. 35734667 PMC9207063

[9] Singh Y, Barua SK, Trivedi S, Tp R, Pratim Kashyap M, Kumar Agrawal L, et al. Skeletal-Related Events in Renal Cell Carcinoma: Prediction With Alkaline Phosphatase (ALP), C-reactive Protein (CRP), Haemoglobin (Hb) and Erythrocyte Sedimentation Rate (ESR) (A.C.H.E.) Score for Risk Stratification. Cureus. 2023;15(6):e40835. Epub 2023/07/25. doi: 10.7759/cureus.40835 ; PubMed Central PMCID: PMC10363263. 37489216 PMC10363263

[10] Liu S, Zhao K, Shao C, Xu L, Cui X, Wang Y. Association between alkaline phosphatase to albumin ratio and mortality among patients with sepsis. Sci Rep. 2024;14(1):3170. Epub 2024/02/08. doi: 10.1038/s41598-024-53384-7 ; PubMed Central PMCID: PMC10850091. 38326383 PMC10850091

[11] Trikudanathan G, Yazici C, Evans Phillips A, Forsmark CE. Diagnosis and Management of Acute Pancreatitis. Gastroenterology. 2024; 167(4):673–688. Epub 2024/ 05/15. doi: 10.1053/j.gastro.2024.02.052 . 38759844

[12] Li Q, Wang H, Wang H, Deng J, Cheng Z, Lin W, et al. Association between serum alkaline phosphatase levels in late pregnancy and the incidence of venous thromboembolism postpartum: a retrospective cohort study. EClinicalMedicine. 2023;62:102088. Epub 2023/08/03. doi: 10.1016/j.eclinm.2023.102088 ; PubMed Central PMCID: PMC10393549. 37533415 PMC10393549

[13] Wang X, Li C, Li M, Zeng X, Mu J, Li Y. Clinical significance of serum lactate and lactate dehydrogenase levels for disease severity and clinical outcomes in patients with colorectal cancer admitted to the intensive care unit. Heliyon. 2024;10(1):e23608. Epub 2024/01/04. doi: 10.1016/j.heliyon.2023.e23608 ; PubMed Central PMCID: PMC10761776. 38173474 PMC10761776

[14] Comandatore A, Franczak M, Smolenski RT, Morelli L, Peters GJ, Giovannetti E. Lactate Dehydrogenase and its clinical significance in pancreatic and thoracic cancers. Semin Cancer Biol. 2022;86(Pt 2):93–100. Epub 2022/09/13. doi: 10.1016/j.semcancer.2022.09.001 . 36096316

[15] Lin H, Yu T, Xu R, Li X. Mean corpuscular hemoglobin predicts the length of hospital stay independent of severity classification in patients with acute pancreatitis. Open Med (Wars). 2022;17(1):1449–54. Epub 2022/09/22. doi: 10.1515/med-2022-0559 ; PubMed Central PMCID: PMC9449682. 36128446 PMC9449682

[16] Pavalean MC, Ionita-Radu F, Jinga M, Costache RS, Balaban DV, Patrasescu M, et al. Ferritin and Ferritin-to-Hemoglobin Ratio as Promising Prognostic Biomarkers of Severity in Acute Pancreatitis-A Cohort Study. Biomedicines. 2024;12(1). Epub 2024/01/23. doi: 10.3390/biomedicines12010106 ; PubMed Central PMCID: PMC10813292. 38255211 PMC10813292

[17] Chen S, Wang L, Gao X, Chen M, Zeng Z, Zhao L. Characterization of serum irisin in patients with severe acute pancreatitis. Eur J Gastroenterol Hepatol. 2019;31(8):985–91. Epub 2019/06/11. doi: 10.1097/MEG.0000000000001454 . 31180990

[18] Baron TH, DiMaio CJ, Wang AY, Morgan KA. American Gastroenterological Association Clinical Practice Update: Management of Pancreatic Necrosis. Gastroenterology. 2020; 158(1):67–75.e1. Epub 2019 Aug 31. doi: 10.1053/j.gastro.2019.07.064 . 31479658

[19] Chen S, Guo S, Gou M, Pan Y, Fan M, Zhang N, et al. A composite indicator of derived neutrophil-lymphocyte ratio and lactate dehydrogenase correlates with outcomes in pancreatic carcinoma patients treated with PD-1 inhibitors. Front Oncol. 2022;12:951985. Epub 2022/11/18. doi: 10.3389/fonc.2022.951985 ; PubMed Central PMCID: PMC9641296. 36387152 PMC9641296

[20] Azevedo B, Fagundes DJ. Differential Diagnosis between Biliary and Nonbiliary Acute Pancreatitis: What Is the Importance of Laboratory Tests? Arq Bras Cir Dig. 2022;35:e1694. Epub 2022/12/01. doi: 10.1590/0102-672020220002e1694 ; PubMed Central PMCID: PMC9704852. 36449864 PMC9704852

[21] Flores-Guerrero JL, Been RA, Shalaurova I, Connelly MA, van Dijk PR, Dullaart RPF. Triglyceride/HDL cholesterol ratio and lipoprotein insulin resistance Score: Associations with subclinical atherosclerosis and incident cardiovascular disease. Clin Chim Acta. 2024;553:117737. Epub 2023/12/25. doi: 10.1016/j.cca.2023.117737 . 38142802

[22] Yang D, Zhao L, Kang J, Wen C, Li Y, Ren Y, et al. Development and validation of a predictive model for acute kidney injury in patients with moderately severe and severe acute pancreatitis. Clin Exp Nephrol. 2022;26(8):770–87. Epub 2022/04/18. doi: 10.1007/s10157-022-02219-8 . 35430680

[23] Yang J, Tang X, Li B, Shi J. Sphingosine 1-phosphate receptor 2 mediated early stages of pancreatic and systemic inflammatory responses via NF-kappa B activation in acute pancreatitis. Cell Commun Signal. 2022; 20(1):157. Epub 2022/10/13. doi: 10.1186/s12964-022-00971-8 . 36229875 PMC9564071

[24] Hong W, Zheng L, Lu Y, Qiu M, Yan Y, Basharat Z, et al. Non-linear correlation between amylase day 2 to day 1 ratio and incidence of severe acute pancreatitis. Front Cell Infect Microbiol. 2022;12:910760. Epub 2022/12/10. doi: 10.3389/fcimb.2022.910760 ; PubMed Central PMCID: PMC9723343. 36483455 PMC9723343

[25] Taneja SK, Mandal R. Attenuation of Zn-induced acute pancreatitis in Wistar rat fed on Cu- and Mg- enriched modified poultry egg Psi. Biol Trace Elem Res. 2008;125(3):245–54. Epub 2008/08/19. doi: 10.1007/s12011-008-8169-9 . 18709337

[26] Su SY, Tang QQ. Altered intestinal microflora and barrier injury in severe acute pancreatitis can be changed by zinc. Int J Med Sci. 2021;18(14):3050–8. Epub 2021/08/18. doi: 10.7150/ijms.45980 ; PubMed Central PMCID: PMC8364456. 34400875 PMC8364456

[27] Xiong X, Rong R, Tang LY, Sun T, Pan YC, Shu HY, et al. The Predictive Value of CA-125 and Hb for Ocular Metastasis in Hepatocellular Carcinoma Patients. Cancer Manag Res. 2022;14:3405–15. Epub 2022/12/13. doi: 10.2147/CMAR.S363115 ; PubMed Central PMCID: PMC9733439. 36504761 PMC9733439

[28] Lu X, Huang X, Xue M, Zhong Z, Wang R, Zhang W, et al. Prognostic significance of increased preoperative red cell distribution width (RDW) and changes in RDW for colorectal cancer. Cancer Med. 2023;12(12):13361–73. Epub 2023/05/05. doi: 10.1002/cam4.6036 ; PubMed Central PMCID: PMC10315724. 37143237 PMC10315724

[29] Yoshida N, Horinouchi T, Toihata T, Harada K, Eto K, Sawayama H, et al. Clinical Significance of Pretreatment Red Blood Cell Distribution Width as a Predictive Marker for Postoperative Morbidity After Esophagectomy for Esophageal Cancer: A Retrospective Study. Ann Surg Oncol. 2022;29(1):606–13. Epub 2021/09/02. doi: 10.1245/s10434-021-10719-2 . 34467503 PMC8407934

[30] Chen K, Cheng X, Xue S, Chen J, Zhang X, Qi Y, et al. Albumin conjugation promotes arsenic trioxide transport through alkaline phosphatase-associated transcytosis in MUC4 wildtype pancreatic cancer cells. Int J Biol Macromol. 2024;257(2):128756. Epub 2023/12/14. doi: 10.1016/j.ijbiomac.2023.128756 . 38092098

[31] Sissingh NJ, Rijk FM, Timmerhuis HC, Umans DS, Anten MF, Bouwense SW, et al. Gallstones as a cause in presumed acute alcoholic pancreatitis: observational multicentre study. Br J Surg. 2024;111(5): e107. Epub 2024/05/03. doi: 10.1093/bjs/znae107 . 38713609

[32] Wang X, Wang C, Zhang H. Improvement of Diagnostic Accuracy for Pancreatic Cancer with Serum Lactate Dehydrogenase. Cancer Manag Res. 2021;13:4879–86. Epub 2021/07/01. doi: 10.2147/CMAR.S312312 ; PubMed Central PMCID: PMC8232858. 34188541 PMC8232858

[33] Dwyre DM, Reddy J, Fernando LP, Donnelly JM, Miller JW, Green R. Microangiopathic thrombocytopenia caused by vitamin B12 deficiency responding to plasma exchange. British journal of haematology. 2024. Epub 2024/07/20 20:43. doi: 10.1111/bjh.19625 . 39030927

[34] Wessely A, Waltera A, Reichert TE, Stöckl S, Grässel S, Bauer RJ. Induction of ALP and MMP9 activity facilitates invasive behavior in heterogeneous human BMSC and HNSCC 3D spheroids. FASEB J.2019;33(11):11884–11893. Epub 2019/07/31. doi: 10.1096/fj.201900925R ; PubMed PMCID: PMC6902691. 31366234 PMC6902691

[35] Chen Y, Li Q, Ma L, Cai Z, Zhou J. Development and internal validation of a practical model to predict 30 days mortality of severe acute pancreatitis patients. Ann Med. 2023; 55 (1):2236648. Epub 2023/12/05. doi: 10.1080/07853890.2023.2236648 ; PubMed PMCID: PMC10364556. 37477658 PMC10364556

[36] Wang J, Li H, Luo H, Shi R, Chen S, Hu J, et al. Association between serum creatinine to albumin ratio and short- and long-term all-cause mortality in patients with acute pancreatitis admitted to the intensive care unit: a retrospective analysis based on the MIMIC-IV database. Front Immunol. 2024;15:1373371. Epub 2024/04/15. doi: 10.3389/fimmu.2024.1373371 ; PubMed PMCID: PMC11056558. 38686375 PMC11056558

[37] Chicco D, Oneto L. Computational intelligence identifies alkaline phosphatase (ALP), alpha-fetoprotein (AFP), and hemoglobin levels as most predictive survival factors for hepatocellular carcinoma. Health Informatics J. 2021;27(1):1460458220984205. Epub 2021/01/29. doi: 10.1177/1460458220984205 . 33504243

